# Supplementary material for: Evaluating open LLMs for agentic analysis orchestration in a typical biomedical lab
Source: bioRxiv. 2026 May 18:2026.05.13.724985. Preprint. [Version 1] doi: 10.64898/2026.05.13.724985 (PMC13228249; doi:10.64898/2026.05.13.724985)
Supplement: Supplement 1 [file NIHPP2026.05.13.724985v1-supplement-1.pdf]

bioRxiv preprint doi: https://doi.org/10.64898/2026.05.13.724985; this version posted May 18, 2026. The copyright holder for this preprint (which was not certified by peer review) is the author/funder, who has granted bioRxiv a license to display the preprint in perpetuity. It is made available under a CC-BY 4.0 International license.

Supplement

Plan files

The full text of each plan file referenced in Table 4. Each plan is reproduced verbatim from `plan/` in the repository and shown inside a fenced Markdown block to preserve its original heading hierarchy and code formatting.

Plan v1 (lean) — `plan/PLAN_v1.md`

```markdown
# Per-sample mtDNA amplicon variant-calling plan

1. **Set globals and prepare results directory**
   - Define `THREADS=4` and the sample list: `M117-bl M117-ch M117C1-bl M117C1-ch`.
   - Create `results/` if missing. Use `set -euo pipefail`.
   - Treat every output step as idempotent: guard each artifact with an existence check (e.g. skip if `results/{sample}.vcf.gz.tbi` already exists and is newer than its inputs). Re-runs on a fully populated `results/` must exit 0 without re-doing work.

2. **Reference indexing (once, in `data/ref/`)**
   - `samtools faidx data/ref/chrM.fa` → produces `chrM.fa.fai`.
   - `bwa index data/ref/chrM.fa` → produces the `.amb .ann .bwt .pac .sa` set.
   - Skip both if the index files already exist.

3. **Per-sample alignment with `bwa mem`**
   - Use `bwa mem -t 4` with the paired FASTQs `data/raw/{sample}_1.fq.gz` and `data/raw/{sample}_2.fq.gz`.
   - Pass the read group via `-R` as a single double-quoted argument containing literal backslash-t between fields and colons between key and value:
     - exact form: `-R "@RG\tID:{sample}\tSM:{sample}\tLB:{sample}\tPL:ILLUMINA"`
     - The `\t` must remain the two characters backslash and `t` - bwa parses them itself. Do NOT use `printf`, `echo -e`, `$'\t'`, or any mechanism that turns them into real tabs; bwa rejects real tabs with "the read group line contained literal <tab> characters".
     - Separators between key and value are colons `:`, not `=`.

4. **SAM → sorted BAM**
   - Pipe `bwa mem` stdout into `samtools sort -@ 4 -o results/{sample}.bam`.
   - Do NOT run `markdup` or `rmdup`: this is amplicon data where PCR duplicates are expected and biologically meaningful.

5. **BAM indexing**
   - `samtools index -@ 4 results/{sample}.bam` → `results/{sample}.bam.bai`.

6. **Variant calling with `lofreq call-parallel`**
   - Use the `call-parallel` subcommand (not plain `lofreq call`) with `--pp-threads 4`.
   - Reference: `data/ref/chrM.fa`. Input: `results/{sample}.bam`.
   - Write uncompressed VCF to a temporary path (e.g. `results/{sample}.vcf`); lofreq emits plain VCF.

7. **VCF compression and indexing**
   - Compress with `bgzip` (not `bcftools view -O z`) producing `results/{sample}.vcf.gz`.
   - Index with `tabix -p vcf results/{sample}.vcf.gz` → `results/{sample}.vcf.gz.tbi`.
   - Remove the intermediate uncompressed `.vcf`.

8. **Collapse step → `results/collapsed.tsv`**
   - For each sample, run `bcftools query -f '{sample}\t%CHROM\t%POS\t%REF\t%ALT\t%INFO/AF\n' results/{sample}.vcf.gz` (the `{sample}` literal is prepended via the format string so the sample name is attached per row).
   - Concatenate all four samples' output.
   - Prepend a single header line `sample\tchrom\tpos\tref\talt\taf` (tab-separated).
   - Output is tab-separated, one variant per line, header on, written to `results/collapsed.tsv`. Rebuild only if any input VCF is newer than the TSV.

9. **Idempotency check**
```

bioRxiv preprint doi: https://doi.org/10.64898/2026.05.13.724985; this version posted May 18, 2026. The copyright holder for this preprint (which was not certified by peer review) is the author/funder, who has granted bioRxiv a license to display the preprint in perpetuity. It is made available under a CC-BY 4.0 International license.

```
   - Final pass: re-running the script on a fully populated `results/` exits 0, performs no work, and leaves
all eight per-sample artifacts plus `collapsed.tsv` intact.
```

Plan v1.25 — plan/PLAN_v1p25.md

```
# Per-sample mtDNA amplicon variant-calling plan

1. **Set globals and prepare results directory**
   - Define `THREADS=4` and the sample list: `M117-bl M117-ch M117C1-bl M117C1-ch`.
   - Create `results/` if missing. Use `set -euo pipefail`.
   - Treat every output step as idempotent: guard each artifact with an existence check (e.g. skip if
`results/{sample}.vcf.gz.tbi` already exists and is newer than its inputs). Re-runs on a fully populated
`results/` must exit 0 without re-doing work.

2. **Reference indexing (once, in `data/ref/`)**
   - `samtools faidx data/ref/chrM.fa` → produces `chrM.fa.fai`.
   - `bwa index data/ref/chrM.fa` → produces the `.amb .ann .bwt .pac .sa` set.
   - Skip both if the index files already exist.

3. **Per-sample alignment with `bwa mem`**
   - Use `bwa mem -t 4` with the paired FASTQs `data/raw/{sample}_1.fq.gz` and `data/raw/{sample}_2.fq.gz`.
   - Pass the read group via `-R` as a single double-quoted argument containing literal backslash-t between
fields and colons between key and value:
     - exact form: `-R "@RG\tID:{sample}\tSM:{sample}\tLB:{sample}\tPL:ILLUMINA"`
     - The `\t` must remain the two characters backslash and `t` - bwa parses them itself. Do NOT use
`printf`, `echo -e`, `$'\t'`, or any mechanism that turns them into real tabs; bwa rejects real tabs with "the
read group line contained literal <tab> characters".
     - Separators between key and value are colons `:`, not `=`.

4. **SAM → sorted BAM**
   - Pipe `bwa mem` stdout into `samtools sort -@ 4 -o results/{sample}.bam`.
   - Do NOT run `markdup` or `rmdup`: this is amplicon data where PCR duplicates are expected and biologically
meaningful.

5. **BAM indexing**
   - `samtools index -@ 4 results/{sample}.bam` → `results/{sample}.bam.bai`.

6. **Variant calling with `lofreq call-parallel`**
   - Exact command:
     ```
     lofreq call-parallel --pp-threads 4 -f data/ref/chrM.fa -o results/{sample}.vcf results/{sample}.bam
     ```
   - Output: `results/{sample}.vcf` (uncompressed). lofreq emits plain VCF.

7. **VCF compression and indexing**
   - Compress with `bgzip` (not `bcftools view -O z`) producing `results/{sample}.vcf.gz`.
   - Index with `tabix -p vcf results/{sample}.vcf.gz` → `results/{sample}.vcf.gz.tbi`.
   - Remove the intermediate uncompressed `.vcf`.

8. **Collapse step → `results/collapsed.tsv`**
   - For each sample, run `bcftools query -f '{sample}\t%CHROM\t%POS\t%REF\t%ALT\t%INFO/AF\n'
results/{sample}.vcf.gz` (the `{sample}` literal is prepended via the format string so the sample name is
attached per row).
   - Concatenate all four samples' output.
   - Prepend a single header line `sample\tchrom\tpos\tref\talt\taf` (tab-separated).
   - Output is tab-separated, one variant per line, header on, written to `results/collapsed.tsv`. Rebuild
only if any input VCF is newer than the TSV.

9. **Idempotency check**
   - Final pass: re-running the script on a fully populated `results/` exits 0, performs no work, and leaves
all eight per-sample artifacts plus `collapsed.tsv` intact.
```

Plan v1.5 — plan/PLAN_v1p5.md

```
# Implementation Plan: Per-sample mtDNA Variant Calling

## Boilerplate (top of `run.sh`)
```

bioRxiv preprint doi: https://doi.org/10.64898/2026.05.13.724985; this version posted May 18, 2026. The copyright holder for this preprint (which was not certified by peer review) is the author/funder, who has granted bioRxiv a license to display the preprint in perpetuity. It is made available under a CC-BY 4.0 International license.

```
set -euo pipefail
THREADS=4
SAMPLES=("M117-bl" "M117-ch" "M117C1-bl" "M117C1-ch")
mkdir -p results
```

All per-sample steps run in `for sample in "${SAMPLES[@]}"; do ... done`.

---

1. Reference indexing - BWA

```
bwa index data/ref/chrM.fa
```

2. Reference indexing - samtools faidx

```
samtools faidx data/ref/chrM.fa
```

3. Per-sample alignment + sort (one pipeline)

```
bwa mem -t 4 -R "@RG\tID:{sample}\tSM:{sample}\tLB:{sample}\tPL:ILLUMINA" data/ref/chrM.fa
data/raw/{sample}_1.fq.gz data/raw/{sample}_2.fq.gz | samtools sort -@ 4 -o results/{sample}.bam -
```

4. BAM index

```
samtools index -@ 4 results/{sample}.bam
```

5. Variant calling - LoFreq

```
lofreq call-parallel --pp-threads 4 -f data/ref/chrM.fa -o results/{sample}.vcf results/{sample}.bam
```

6. VCF compression + tabix index

```
bgzip -f results/{sample}.vcf
```
```
tabix -p vcf results/{sample}.vcf.gz
```

7. Collapsed TSV

```
printf 'sample\tchrom\tpos\tref\talt\taf\n' > results/collapsed.tsv
```
```
bcftools query -f '%CHROM\t%POS\t%REF\t%ALT\t%INFO/AF\n' results/{sample}.vcf.gz | awk -v s={sample}
'BEGIN{OFS="\t"}{print s,$0}' >> results/collapsed.tsv
```

Plan v1g — plan/PLAN_v1g.md

Per-sample mtDNA amplicon variant-calling plan
<!--
v1g = v1 with Galaxy-IUC-derived CLI snippets injected per step where IUC has
clean coverage. Extracted mechanically by scripts/galaxy_to_snippet.py from

bioRxiv preprint doi: https://doi.org/10.64898/2026.05.13.724985; this version posted May 18, 2026. The copyright holder for this preprint (which was not certified by peer review) is the author/funder, who has granted bioRxiv a license to display the preprint in perpetuity. It is made available under a CC-BY 4.0 International license.

```
tools-iuc commit 39e745658a6ff7f013788871916574117a0f47f1 (2026-04-27).

IUC coverage map:
  bwa, bwa-mem        : extraction yields mostly noise (heavy macro use) - fallback to v1 prose
  samtools_faidx       : all-conditional command block - fallback to v1 prose
  samtools_sort        : partial extraction with placeholders - fallback to v1 prose
  samtools_index       : not in IUC - fallback to v1 prose
  lofreq_call_parallel : clean extraction - INJECTED (step 6)
  bgzip / tabix        : not in IUC - fallback to v1 prose
  bcftools_query       : format string in stripped Cheetah var - fallback to v1 prose
-->

1. **Set globals and prepare results directory**
   - Define `THREADS=4` and the sample list: `M117-bl M117-ch M117C1-bl M117C1-ch`.
   - Create `results/` if missing. Use `set -euo pipefail`.
   - Treat every output step as idempotent: guard each artifact with an existence check (e.g. skip if
`results/{sample}.vcf.gz.tbi` already exists and is newer than its inputs). Re-runs on a fully populated
`results/` must exit 0 without re-doing work.

2. **Reference indexing (once, in `data/ref/`)**
   - `samtools faidx data/ref/chrM.fa` → produces `chrM.fa.fai`.
   - `bwa index data/ref/chrM.fa` → produces the `.amb .ann .bwt .pac .sa` set.
   - Skip both if the index files already exist.

3. **Per-sample alignment with `bwa mem`**
   - Use `bwa mem -t 4` with the paired FASTQs `data/raw/{sample}_1.fq.gz` and `data/raw/{sample}_2.fq.gz`.
   - Pass the read group via `-R` as a single double-quoted argument containing literal backslash-t between
fields and colons between key and value:
     - exact form: `-R "@RG\tID:{sample}\tSM:{sample}\tLB:{sample}\tPL:ILLUMINA"`
     - The `\t` must remain the two characters backslash and `t` - bwa parses them itself. Do NOT use
`printf`, `echo -e`, `$'\t'`, or any mechanism that turns them into real tabs; bwa rejects real tabs with "the
read group line contained literal <tab> characters".
     - Separators between key and value are colons `:`, not `=`.

4. **SAM → sorted BAM**
   - Pipe `bwa mem` stdout into `samtools sort -@ 4 -o results/{sample}.bam`.
   - Do NOT run `markdup` or `rmdup`: this is amplicon data where PCR duplicates are expected and biologically
meaningful.

5. **BAM indexing**
   - `samtools index -@ 4 results/{sample}.bam` → `results/{sample}.bam.bai`.

6. **Variant calling with `lofreq call-parallel`**
   - Galaxy IUC canonical invocation (extracted from `tools/lofreq/lofreq_call.xml` @ tools-iuc 39e7456):
     ```
     lofreq call-parallel --pp-threads 4 --verbose
     --ref data/ref/chrM.fa --out results/{sample}.vcf
     --sig
     --bonf
     results/{sample}.bam
     ```
     (The bare `--sig` and `--bonf` lines come from Galaxy-runtime-supplied values; you can omit them and use
lofreq's defaults. The load-bearing detail is that `results/{sample}.bam` is a **positional argument at the
end**, not behind `-i`/`-b`/`-bam`.)

7. **VCF compression and indexing**
   - Compress with `bgzip` (not `bcftools view -O z`) producing `results/{sample}.vcf.gz`.
   - Index with `tabix -p vcf results/{sample}.vcf.gz` → `results/{sample}.vcf.gz.tbi`.
   - Remove the intermediate uncompressed `.vcf`.

8. **Collapse step → `results/collapsed.tsv`**
   - For each sample, run `bcftools query -f '{sample}\t%CHROM\t%POS\t%REF\t%ALT\t%INFO/AF\n'
results/{sample}.vcf.gz` (the `{sample}` literal is prepended via the format string so the sample name is
attached per row).
   - Concatenate all four samples' output.
   - Prepend a single header line `sample\tchrom\tpos\tref\talt\taf` (tab-separated).
   - Output is tab-separated, one variant per line, header on, written to `results/collapsed.tsv`. Rebuild
only if any input VCF is newer than the TSV.
```

bioRxiv preprint doi: https://doi.org/10.64898/2026.05.13.724985; this version posted May 18, 2026. The copyright holder for this preprint (which was not certified by peer review) is the author/funder, who has granted bioRxiv a license to display the preprint in perpetuity. It is made available under a CC-BY 4.0 International license.

````
9. **Idempotency check**
   - Final pass: re-running the script on a fully populated `results/` exits 0, performs no work, and leaves all eight per-sample artifacts plus `collapsed.tsv` intact.
````

Plan v2 (detailed) — `plan/PLAN.md`

````
# Implementation Plan: Per-sample mtDNA Variant Calling

## Boilerplate (top of `run.sh`)
- First line after shebang: `set -euo pipefail`.
- Constants: `THREADS=4` and `SAMPLES=("M117-bl" "M117-ch" "M117C1-bl" "M117C1-ch")`.
- Create output dir: `mkdir -p results`.
- All per-sample steps must be wrapped in `for sample in "${SAMPLES[@]}"; do ... done`.

---

## 1. Reference indexing - BWA

```
bwa index data/ref/chrM.fa
```

- Outputs (5 sibling files): `data/ref/chrM.fa.amb`, `.ann`, `.bwt`, `.pac`, `.sa`.
- Idempotency guard: `[[ -f data/ref/chrM.fa.bwt ]] || bwa index data/ref/chrM.fa`
- Gotcha: `bwa index` writes outputs next to the input; the dir must be writable. No flags needed for a 16 kb reference (default algorithm is fine).

## 2. Reference indexing - samtools faidx

```
samtools faidx data/ref/chrM.fa
```

- Output: `data/ref/chrM.fa.fai`.
- Guard: `[[ -f data/ref/chrM.fa.fai ]] || samtools faidx data/ref/chrM.fa`

## 3. Per-sample alignment + sort (one pipeline)

```
bwa mem -t 4 -R "@RG\tID:{sample}\tSM:{sample}\tLB:{sample}\tPL:ILLUMINA" data/ref/chrM.fa
data/raw/{sample}_1.fq.gz data/raw/{sample}_2.fq.gz | samtools sort -@ 4 -o results/{sample}.bam -
```

- Output: `results/{sample}.bam`.
- Guard: `[[ -f results/{sample}.bam ]] || { bwa mem ... | samtools sort ... ; }` - wrap the whole pipeline in braces so the guard covers both stages.
- RG string gotchas (CRITICAL):
  - Use colons (`ID:`, `SM:`, `LB:`, `PL:`) - never `=`.
  - Use the **literal two characters** `\t` (backslash + t) inside the double-quoted string. Do NOT use `printf`, `echo -e`, `$'\t'`, or a real tab. `bwa` expands `\t` itself; a real tab corrupts the SAM header.
  - The whole `-R` value must be a single double-quoted argument.
- `samtools sort` trailing `-` reads from stdin.

## 4. BAM index

```
samtools index -@ 4 results/{sample}.bam
```

- Output: `results/{sample}.bam.bai`.
- Guard: `[[ -f results/{sample}.bam.bai ]] || samtools index -@ 4 results/{sample}.bam`
- Do NOT run `markdup` - this is amplicon data; PCR duplicates are expected and informative.

## 5. Variant calling - LoFreq

```
lofreq call-parallel --pp-threads 4 -f data/ref/chrM.fa -o results/{sample}.vcf results/{sample}.bam
````
````

bioRxiv preprint doi: https://doi.org/10.64898/2026.05.13.724985; this version posted May 18, 2026. The copyright holder for this preprint (which was not certified by peer review) is the author/funder, who has granted bioRxiv a license to display the preprint in perpetuity. It is made available under a CC-BY 4.0 International license.

```

- Output: `results/{sample}.vcf` (uncompressed).
- Guard: `[[ -f results/{sample}.vcf || -f results/{sample}.vcf.gz ]] || lofreq call-parallel --pp-threads 4 -f data/ref/chrM.fa -o results/{sample}.vcf results/{sample}.bam`
  (Check both because step 6 will delete the `.vcf` and leave `.vcf.gz`.)
- Gotchas: BAM is positional, NOT behind `-b`/`-i`. The flag is `--pp-threads`, not `-t` or `--threads`. Reference (`-f`) requires the `.fai` from step 2 to already exist.

## 6. VCF compression + tabix index

```
bgzip -f results/{sample}.vcf
```
```
tabix -p vcf results/{sample}.vcf.gz
```

- Outputs: `results/{sample}.vcf.gz` and `results/{sample}.vcf.gz.tbi`.
- Combined guard: `[[ -f results/{sample}.vcf.gz.tbi ]] || { bgzip -f results/{sample}.vcf && tabix -p vcf results/{sample}.vcf.gz ; }`
- Gotchas: `bgzip` operates **in place** - it deletes `results/{sample}.vcf` after writing `.vcf.gz`. `-f` overwrites any stale `.vcf.gz`. `tabix -p vcf` sets the preset for VCF coordinates.

## 7. Collapsed TSV (rebuild every run)

Do NOT guard this step - always overwrite, since per-sample VCFs may have changed.

Header (overwrite):

```
printf 'sample\tchrom\tpos\tref\talt\taf\n' > results/collapsed.tsv
```

Per sample, append:

```
bcftools query -f '%CHROM\t%POS\t%REF\t%ALT\t%INFO/AF\n' results/{sample}.vcf.gz | awk -v s={sample}
'BEGIN{OFS="\t"}{print s,$0}' >> results/collapsed.tsv
```

- Gotchas:
  - The format string uses `%INFO/AF`, not `%AF` - bcftools requires the `INFO/` prefix for INFO fields.
  - The `\t` and `\n` inside `-f '...'` are **bcftools format codes**, parsed by bcftools itself; keep them inside single quotes so the shell doesn't touch them.
  - awk's `OFS="\t"` is required so `print s,$0` joins with a tab (`$0` already contains the tabbed bcftools row, so the result is `sample<TAB>chrom<TAB>pos<TAB>ref<TAB>alt<TAB>af`).
  - Use `>` for the header line, `>>` for every per-sample append.

Idempotency summary
- Steps 1-6 each have a `[[ -f <sentinel> ]] ||` guard on their final output. A second invocation on a populated `results/` performs no alignment, calling, compression, or indexing work.
- Step 7 is intentionally rebuilt from scratch on every run (header `>`, then append per sample). This is cheap (one `bcftools query` per sample) and prevents stale rows if any VCF changed. Exit status of a fully-cached run is `0`.

Plan v2_defensive — plan/PLAN_v2_defensive.md

`run.sh` Implementation Plan - chrM amplicon variant calling

0. Script preamble (top of file)

1. First line after shebang: `set -euo pipefail`.
2. Constants: `THREADS=4` and `SAMPLES=("M117-bl" "M117-ch" "M117C1-bl" "M117C1-ch")`.
3. Paths: `REF=data/ref/chrM.fa`, `OUT=results`.
4. `mkdir -p "$OUT"`.
```

bioRxiv preprint doi: https://doi.org/10.64898/2026.05.13.724985; this version posted May 18, 2026. The copyright holder for this preprint (which was not certified by peer review) is the author/funder, who has granted bioRxiv a license to display the preprint in perpetuity. It is made available under a CC-BY 4.0 International license.

5. Initialize the failure log every run (truncate, no header - pure TSV body): `: > "$OUT/failures.log"`.
6. Track survivors with an array: `SURVIVORS=()` and a counter `OK=0`.

0a. Defensive helper `try`

Define this function exactly:

```
try() {  # try <sample> <step_label> <validation_cmd_string> -- <cmd...>
  local sample="$1" step="$2" validate="$3"; shift 3
  [[ "$1" == "--" ]] && shift
  if "$@" && eval "$validate"; then return 0; fi
  "$@" && eval "$validate" && return 0
  printf '%s\t%s\t%s\n' "$sample" "$step" "command_or_validation_failed" >> "$OUT/failures.log"
  return 1
}
```

Behavior: runs `cmd`, then evaluates the validation string; on any failure retries the **same cmd + same validation** exactly once; on second failure appends one TSV row to `results/failures.log` and returns 1. Callers must use `if ! try ...; then continue; fi` inside the per-sample loop so one bad sample does **not** abort the script (`set -e` is bypassed because `try` is in an `if` test).

For reference-prep steps (no sample), use sample label `__ref__` and `exit 1` instead of `continue` on failure.

---

1. Reference preparation (once, before the sample loop)

1a. `bwa index`

```
bwa index data/ref/chrM.fa
```

- Outputs: `data/ref/chrM.fa.{amb,ann,bwt,pac,sa}`.
- Idempotency guard: `[[ -f data/ref/chrM.fa.bwt ]] || try __ref__ bwa_index '[[ -s data/ref/chrM.fa.bwt ]]' -- bwa index data/ref/chrM.fa`
- On failure after retry: `echo "[run.sh] reference index failed" >&2; exit 1`.

1b. `samtools faidx`

```
samtools faidx data/ref/chrM.fa
```

- Output: `data/ref/chrM.fa.fai`.
- Guard: `[[ -f data/ref/chrM.fa.fai ]] || try __ref__ faidx '[[ -s data/ref/chrM.fa.fai ]]' -- samtools faidx data/ref/chrM.fa`
- On failure: `exit 1`.

---

2. Per-sample loop

`for s in "${SAMPLES[@]}"; do ... done`. Inside the loop, every `try` failure must `continue` to the next sample. If the current step is skipped by its idempotency guard AND its output validates, fall through; otherwise `continue`.

Step 2a - Align + sort → `results/{s}.bam`

Exact pipeline (the RG string must be a literal double-quoted string containing the four characters `\`, `t`; do **not** use `printf`, `echo -e`, or `$'\t'` - bwa parses `\t` itself):

```
bwa mem -t 4 -R "@RG\tID:${s}\tSM:${s}\tLB:${s}\tPL:ILLUMINA" \
  data/ref/chrM.fa data/raw/${s}_1.fq.gz data/raw/${s}_2.fq.gz \
```

bioRxiv preprint doi: https://doi.org/10.64898/2026.05.13.724985; this version posted May 18, 2026. The copyright holder for this preprint (which was not certified by peer review) is the author/funder, who has granted bioRxiv a license to display the preprint in perpetuity. It is made available under a CC-BY 4.0 International license.

```
  | samtools sort -@ 4 -o results/${s}.bam -
```

- Output: `results/${s}.bam`.
- Guard: `[[ -f results/${s}.bam ]] && samtools quickcheck results/${s}.bam` → skip; else run via `try`.
- Validation string passed to `try`: `'samtools quickcheck results/'"$s"'.bam'`.
- Wrap the whole pipeline in a tiny inline shell function or `bash -c` because `try` takes argv, not a pipeline. Recommended pattern: define `align_one() { bwa mem ... | samtools sort ... ; }` inside the loop, then `try "$s" align '...validation...' -- align_one`.
- Gotchas: literal `\t` only; PL is `ILLUMINA` (uppercase); `samtools sort`'s trailing `-` reads stdin; `-o` precedes the input dash.

Step 2b - BAM index → `results/{s}.bam.bai`

```
samtools index -@ 4 results/${s}.bam
```

- Output: `results/${s}.bam.bai`.
- Guard: `[[ -s results/${s}.bam.bai ]]` → skip.
- Validation: `'[[ -s results/'"$s"'.bam.bai ]]'`.
- No duplicate marking (amplicon data).

Step 2c - Variant calling → `results/{s}.vcf`

Exact command (no substitutions, no extra flags):

```
lofreq call-parallel --pp-threads 4 -f data/ref/chrM.fa -o results/${s}.vcf results/${s}.bam
```

- Output: `results/${s}.vcf` (uncompressed; lofreq writes plain VCF here).
- Guard: skip if `results/${s}.vcf.gz` already exists and tabix index validates (step 2d covers it); otherwise if `results/${s}.vcf` exists and is structurally valid, skip.
- Validation: `'[[ -s results/'"$s"'.vcf ]] && bcftools view -h results/'"$s"'.vcf > /dev/null'`.
- Gotcha: `--pp-threads` is mandatory for `call-parallel`; `-t` is wrong here.

Step 2d - Compress + tabix → `results/{s}.vcf.gz` + `.tbi`

Two separate invocations, each wrapped in its own `try`:

```
bgzip -f results/${s}.vcf
tabix -p vcf results/${s}.vcf.gz
```

- Outputs: `results/${s}.vcf.gz`, `results/${s}.vcf.gz.tbi`.
- Guard for the pair: `[[ -s results/${s}.vcf.gz && -s results/${s}.vcf.gz.tbi ]] && bcftools view -h results/${s}.vcf.gz > /dev/null` → skip both.
- Validation after `bgzip`: `'[[ -s results/'"$s"'.vcf.gz ]] && bcftools view -h results/'"$s"'.vcf.gz > /dev/null'`.
- Validation after `tabix`: `'[[ -s results/'"$s"'.vcf.gz.tbi ]]'`.
- Gotcha: `bgzip -f` **deletes** `results/${s}.vcf` on success - that is expected; do not look for it afterwards.

Step 2e - Mark survivor

After all four steps succeed for sample `s`: `SURVIVORS+=("$s"); OK=$((OK+1))`.

---

3. Collapsed TSV (after the loop, only over `SURVIVORS`)

1. Write header (always, overwrite):

```
printf 'sample\tchrom\tpos\tref\talt\taf\n' > results/collapsed.tsv
```

bioRxiv preprint doi: https://doi.org/10.64898/2026.05.13.724985; this version posted May 18, 2026. The copyright holder for this preprint (which was not certified by peer review) is the author/funder, who has granted bioRxiv a license to display the preprint in perpetuity. It is made available under a CC-BY 4.0 International license.

2. For each `s` in `"${SURVIVORS[@]}"`:

```
bcftools query -f '%CHROM\t%POS\t%REF\t%ALT\t%INFO/AF\n' results/${s}.vcf.gz \
  | awk -v s=${s} 'BEGIN{OFS="\t"}{print s,$0}' >> results/collapsed.tsv
```

Wrap in `try "$s" collapse '[[ -s results/collapsed.tsv ]]' -- ...` so a single broken VCF only loses that row.

---

4. Final summary + exit code

1. Build a comma-separated list of failed samples by `cut -f1 results/failures.log | sort -u | grep -v '^__ref__$' | paste -sd,`.
2. Identify the first failing step per failed sample (e.g. `awk -F'\t' '!seen[$1]++{print $1" failed at "$2}' results/failures.log`).
3. Emit on **stderr**, as the very last line:

```
[run.sh] <OK>/<TOTAL> samples completed; <sample> failed at step <label> - see results/failures.log
```

If `OK == ${#SAMPLES[@]}`: `[run.sh] 4/4 samples completed; no failures`.

4. Exit policy: `if (( OK >= 1 )); then exit 0; else exit 1; fi`.

---

5. Idempotency note

Every step is guarded by an `[[ -f ... ]] && validation` check before invoking `try`. A second run on a fully populated `results/` performs zero tool invocations, re-truncates `results/failures.log` to empty, rewrites `results/collapsed.tsv` from the existing `*.vcf.gz`, and exits 0 with summary `4/4 samples completed; no failures`.
